# Erythrocytes as immunomodulators in trauma: orchestrating clearance pathways, inflammation, and organ injury

**DOI:** 10.3389/fimmu.2026.1909329

**Published:** 2026-09-03

**Authors:** Xuru Qian, Linglong Chen, Renxian Gao, Yue Lin, Kaihuai Wu, Xiaojie Wang, Yingying Jiang

**Affiliations:** 1 Department of Emergency, Wenzhou Third Clinical Institute Affiliated to Wenzhou Medical University, Wenzhou People’s Hospital, The Wenzhou People’s Hospital Affiliated of Hangzhou Medical College, Wenzhou, China; 2 Department of Infectious Diseases, Taishun People’s Hospital, Wenzhou, China

**Keywords:** acute traumatic injury, damage-associated molecular patterns (DAMPs), erythrocytes, immune clearance, immunomodulation, inflammatory response

## Abstract

Acute traumatic injury triggers complex systemic inflammatory and immune responses and remains a leading global cause of mortality and disability. Traditionally viewed primarily as passive oxygen carriers, erythrocytes are now emerging as key participants with significant immunomodulatory functions in the post-traumatic milieu. This narrative review presents a conceptual framework for understanding the evolving and dual-faceted role of erythrocytes in immune clearance pathways following acute injury. We focus on the known and hypothesized mechanisms by which erythrocytes actively modulate inflammatory responses, participate in clearing damage-associated substances, and influence tissue repair and systemic outcomes-processes achieved through surface molecule remodeling, the release of damage-associated molecular patterns (DAMPs), and dynamic interactions with immune cells. Importantly, we highlight that erythrocytes exert beneficial roles in homeostasis (e.g., regulated phagocytosis and immune complex shuttling) but can also become drivers of pathology when their clearance and activation pathways are dysregulated in the traumatic context. By integrating evidence from trauma patients, relevant animal models, and analogous pathological states, this review examines and synthesizes the novel and increasingly recognized roles of erythrocytes in traumatic immunopathology and provides a theoretical foundation for developing novel therapeutic strategies targeting erythrocyte function to improve patient outcomes.

## Highlights

- Paradigm shift: Erythrocytes are not passive oxygen carriers but active immunomodulators that sense and respond to traumatic injury.
- Dual roles: RBCs mediate beneficial homeostatic functions (clearance of debris, immune complex transport) but can also drive maladaptive thrombo-inflammation, organ injury, and immunosuppression when dysregulated.
- Mechanistic network: Key pathways include surface molecule remodeling (PS/CD47), DAMP release (heme/Hb), and crosstalk with phagocytes, complement, platelets, and endothelium.
- Translational potential: RBC-derived biomarkers (e.g., cell-free heme, arginase-1) and RBC-targeted therapies (e.g., complement inhibitors, drug delivery vehicles) represent emerging opportunities for precision management in trauma.
- Critical gaps: Direct evidence from trauma patients remains limited; most mechanistic insights derive from animal models or analogous disease states, highlighting the need for human translational studies.

## 1 Introduction

Acute traumatic injury initiates a profound and complex pathophysiological cascade characterized by immediate tissue damage, hemorrhage, and subsequent ischemia-reperfusion injury, culminating in a potent systemic inflammatory response syndrome (SIRS). This initial insult triggers massive cellular injury and death, leading to the release of a multitude of endogenous damage-associated molecular patterns (DAMPs) that potently activate the innate immune system. Within this chaotic early phase, circulating red blood cells (RBCs) are not merely the primary cellular component lost during hemorrhage; they are among the first cells to encounter the hostile injury microenvironment and undergo significant alterations. Far from being passive victims, emerging evidence reveals that post-traumatic RBCs actively participate in shaping the host’s immune response. They achieve this through mechanisms such as alterations in surface antigen expression, the release of bioactive substances, and direct interactions with immune cells, thereby critically influencing the immune clearance pathways (1). This active regulatory role profoundly impacts the trajectory of the subsequent inflammatory response, the development of secondary organ injury, and ultimately, the clinical outcome. The concept of “blood failure” in trauma underscores the intricate interplay between the oxygen debt incurred from hemorrhage, the endotheliopathy of trauma (EoT), and acute traumatic coagulopathy (ATC), where RBCs are central players (2). Managing this triad requires a multifaceted resuscitation strategy, often involving aggressive transfusion of blood products, including RBCs, to repay oxygen debt and address coagulation dysfunction (2).

The role of RBCs extends beyond oxygen carriage; they are now recognized as important modulators of the immune landscape following injury. In patients with severe thermal and major traumatic injury, markedly elevated plasma concentrations of total heme, primarily released from lysed RBCs and injured muscle, are observed. This heme acts as a potent DAMP with immunomodulatory properties (3). Elevated heme levels correlate with poor clinical outcomes, including increased mortality and sepsis, and are associated with systemic immune suppression. Mechanistically, heme contributes to the development of endotoxin tolerance, as evidenced by reduced lipopolysaccharide (LPS)-induced tumor necrosis factor-alpha (TNF-α) production by leukocytes from injured patients and *in vitro* heme-treated monocytes (3). This immunosuppressive effect may be a double-edged sword, potentially limiting excessive inflammation but also increasing susceptibility to infections. Furthermore, RBC-derived factors may influence tissue catabolism and secondary injury in a context-dependent manner. For example, in traumatic hemarthrosis, RBCs themselves do not directly drive meniscus degradation, whereas in acute brain injury, RBC lysate exposure induces astrocytic pro-inflammatory responses (4, 5). These observations illustrate that the impact of RBC breakdown products varies across tissue types.

Anemia is a common consequence of major trauma due to blood loss and is independently associated with worse outcomes, particularly in patients with acute brain injuries such as traumatic brain injury (TBI), aneurysmal subarachnoid hemorrhage (aSAH), or intracranial hemorrhage (ICH) (6). The management of post-traumatic anemia through RBC transfusion remains debated. In patients with acute brain injury, randomized controlled trials suggest that a liberal transfusion strategy (hemoglobin threshold 9–10 g/dL) may improve neurological outcomes compared with restrictive approaches (6, 7). This benefit may relate not only to improved oxygen delivery but also to avoidance of crystalloid overload that could exacerbate intracranial hypertension (7). However, transfusion carries risks: increased platelet:RBC ratios are associated with acute kidney injury in children, and perioperative transfusion correlates with higher rates of flap necrosis and infection in lower extremity reconstruction (8, 9). These findings underscore the complex risk-benefit profile of RBC transfusion following trauma.

The genetic predisposition of an individual may also influence RBC-related phenotypes and responses in critical illness. Studies on phenotype-genotype correlations in patients with chronic traumatic brain injury and acute COVID-19 have shown that the cumulative effects of rare genetic variants in specific gene sets, including those involved in the Notch signaling pathway, are associated with anemia and alterations in RBC indices in both chronic and acute severe health conditions (10). This suggests that genetic variability contributes to how RBCs perform under physiological stress, potentially affecting an individual’s resilience or susceptibility to the hematological consequences of trauma.

The unique biophysical and immunological properties of RBCs have also inspired novel therapeutic and diagnostic platforms. The RBC membrane possesses distinctive attributes—including prolonged circulation and immune evasion via CD47—that have inspired novel therapeutic platforms (1, 11). RBC membrane-coated nanoparticles are being developed to improve drug delivery and targeting (12–14), while anti-CD47 antibodies are being explored to enhance hematoma clearance after intracerebral hemorrhage (15). Additionally, RBC-based platforms have been applied in antigen-specific immune tolerance induction and diagnostic contexts (16, 17).

It is important to note that many of these mechanistic insights are derived from disease states beyond trauma. The interplay between RBCs and the immune system is also evident in infectious contexts. For example, during malaria, Plasmodium-infected RBCs can impair host antibacterial innate immunity through mechanisms involving hemozoin-bound bioactive molecules, increasing susceptibility to co-infections (18). Similarly, splenic red pulp macrophages play an important role in clearing liver-resistant encapsulated bacteria like Streptococcus pneumoniae from the bloodstream (19). While these observations from non-trauma conditions provide valuable mechanistic analogies, their direct translation to the post-traumatic setting requires cautious interpretation and further investigation.

This is a narrative review that synthesizes literature published up to June 2026. We conducted a structured literature search in PubMed and Web of Science using combinations of keywords including “erythrocytes,” “red blood cells,” “trauma,” “injury,” “immune clearance,” “DAMPs,” “hemolysis,” “phagocytosis,” “coagulation,” and “organ injury.” We prioritized studies on traumatic injury but also drew mechanistic insights from related fields (e.g., hemolytic disorders, sepsis, extracorporeal circulation) where trauma-specific data were lacking. The final reference list was curated to balance foundational mechanistic discoveries with recent advances. Throughout the manuscript, we explicitly indicate the evidence base for each mechanism discussed—whether derived from direct trauma patient data, trauma animal models, or analogous pathological states—to provide readers with a clear understanding of the strength and limitations of the current evidence.

## 2 Post-traumatic erythrocyte alterations: from phenotype to function

### 2.1 Surface molecular remodeling and the regulation of phagocytic clearance

#### 2.1.1 Phosphatidylserine externalization and eryptosis

Externalization of phosphatidylserine (PS) on the erythrocyte membrane serves as a recognition ligand for phagocytic clearance (20). This process, termed eryptosis, represents a form of programmed erythrocyte death that occurs in response to various stressors, including oxidative injury, calcium overload, and energy depletion. Under homeostatic conditions, PS exposure ensures the orderly removal of senescent erythrocytes by macrophages in the spleen and liver (20). In the post-traumatic context, mechanical shear stress from hemorrhage and resuscitation, along with ischemia-reperfusion-induced oxidative injury, may induce PS exposure on a substantial proportion of circulating erythrocytes (21). While direct evidence from trauma patients is limited, studies in extracorporeal life support (ECLS)—a setting involving analogous mechanical erythrocyte stress—demonstrate significantly increased PS-positive erythrocytes and accelerated clearance, contributing to net erythrocyte loss (21). It is hypothesized, though not yet confirmed in human trauma cohorts, that excessive PS exposure could saturate the phagocytic capacity of the reticuloendothelial system, potentially impairing the clearance of pathogens and cellular debris and thereby contributing to immune dysfunction.

#### 2.1.2 CD47-SIRPα signaling in phagocytic regulation

The phagocytic clearance of PS-exposed erythrocytes is modulated by inhibitory signaling pathways, of which the CD47-SIRPα axis is a well-characterized example. CD47 on the erythrocyte surface engages SIRPα on macrophages, transmitting an inhibitory signal that suppresses phagocytosis of viable cells (22). In experimental models of cellular stress, loss or downregulation of CD47 expression on damaged erythrocytes facilitates their recognition and removal (23). Conversely, sustained CD47 expression on pathological erythrocytes may permit their persistence in circulation, as demonstrated in polycythemia models where disruption of the CD47-SIRPα axis reverses the disease phenotype (24). In the context of trauma, scald injury models suggest that targeting this axis may mitigate T-cell dysfunction (23). However, whether CD47 modulation constitutes a significant determinant of erythrocyte clearance in human trauma—and whether its dysregulation contributes to either excessive clearance of viable erythrocytes or persistence of pro-inflammatory cell populations—remains an open question that requires direct investigation in trauma cohorts.

#### 2.1.3 Complement regulatory proteins and adhesion molecules

Beyond PS and CD47, erythrocytes express a repertoire of complement regulatory proteins, including CD35 (complement receptor 1), CD55 (decay-accelerating factor), and CD59 (membrane inhibitor of reactive lysis), which under physiological conditions protect host cells from inadvertent complement attack (25). Dysregulation of these proteins on erythrocytes may predispose to increased deposition of complement components, including the membrane attack complex C5b-9, promoting intravascular hemolysis and the release of DAMPs (26). While this mechanism is well documented in conditions such as transplant-associated thrombotic microangiopathy, whether similar alterations occur on erythrocytes following trauma remains unexplored.

In addition, trauma-induced stress may upregulate adhesion molecules such as ICAM-4 on erythrocytes, enhancing their interaction with endothelial cells, platelets, and immune cells. This adhesive phenotype can promote erythrocyte aggregation and adherence to the vascular wall, potentially impeding microvascular flow and exacerbating tissue ischemia (27). In cerebral malaria, this mechanism constitutes a central pathophysiological event leading to microvascular obstruction (27). Its relevance to trauma is suggested by acute subdural hematoma models, in which corpuscular blood components—including erythrocytes—drive neuroinflammation and blood-brain barrier breakdown, processes likely facilitated by adhesive interactions (28).

### 2.2 Release and function of damage-associated molecular patterns

#### 2.2.1 Direct release of DAMPs from injured erythrocytes

Intravascular hemolysis, a frequent consequence of traumatic injury, releases hemoglobin and its breakdown product heme, which function as DAMPs with pleiotropic immunomodulatory effects. Free hemoglobin scavenges nitric oxide, promoting vasoconstriction and endothelial dysfunction, while heme activates Toll-like receptor 4 (TLR4), initiating pro-inflammatory signaling cascades (28, 29). In trauma patients, elevated plasma heme levels correlate with poor clinical outcomes and systemic immune suppression (3), a relationship mechanistically supported by acute subdural hematoma models in which lysed erythrocyte components drive neuroinflammation via TLR4-dependent cytokine production (28). Beyond proteinaceous DAMPs, erythrocytes under stress release nucleotides such as ATP, which activates purinergic receptors (e.g., P2X7) on immune cells, regulating inflammasome activation and cytokine release (30). Cell-free mitochondria have also been detected in trauma patient plasma and may represent a distinct category of DAMP, though their erythrocyte-specific origin requires clarification.

#### 2.2.2 Vesicle-mediated signal propagation and redox amplification

Erythrocyte-derived extracellular vesicles—including microvesicles and exosomes—are shed from the erythrocyte membrane in response to stress or injury. These vesicles carry proteins, lipids, and nucleic acids that reflect their cell of origin, and function as intercellular communication vehicles, transferring bioactive signals to endothelial cells, immune cells, and distant organs (21). In extracorporeal life support—a setting involving mechanical erythrocyte stress analogous to that in trauma—vesicle levels are markedly elevated and correlate with erythrocyte activation and injury (21). The release of erythrocyte-derived DAMPs and vesicles may establish a feed-forward cycle: oxidative stress damages erythrocyte membranes, promoting further hemolysis and DAMP release, while reactive oxygen species generated from hemoglobin metabolism amplify tissue injury (29, 31). This cycle is hypothesized to contribute to secondary organ injury, as exemplified by the association between hemolysis biomarkers and acute kidney injury in rhabdomyolysis (32, 33). While direct evidence for this redox cycle in trauma patients remains limited, this framework provides a plausible mechanistic link between erythrocyte injury and remote organ dysfunction that warrants investigation in trauma cohorts.

## 3 Regulation of erythrocyte-mediated immune clearance pathways

### 3.1 Modulation of phagocyte function

Erythrophagocytosis by splenic and hepatic macrophages modulates the functional state of these resident cells (34). In sterile inflammation models, hemoglobin- or heme-activated macrophages polarize toward a pro-inflammatory phenotype, whereas erythrophagocytosis induces an anti-inflammatory phenotype characterized by HMOX1 and MARCO expression (35). This plasticity, driven by transcription factors such as NFE2L2/NRF2, links erythrocyte clearance to innate immune regulation.

The CD47-SIRPα axis, which suppresses phagocytosis of viable erythrocytes, represents a key checkpoint in this process (36). In the trauma setting, transfusion of stored erythrocytes may impair macrophage function. Using a murine model of trauma and hemorrhagic shock, supernatant from stored human erythrocytes inhibited macrophage clearance of Escherichia coli in an HMGB1-dependent manner (37).

**Figure 1:**
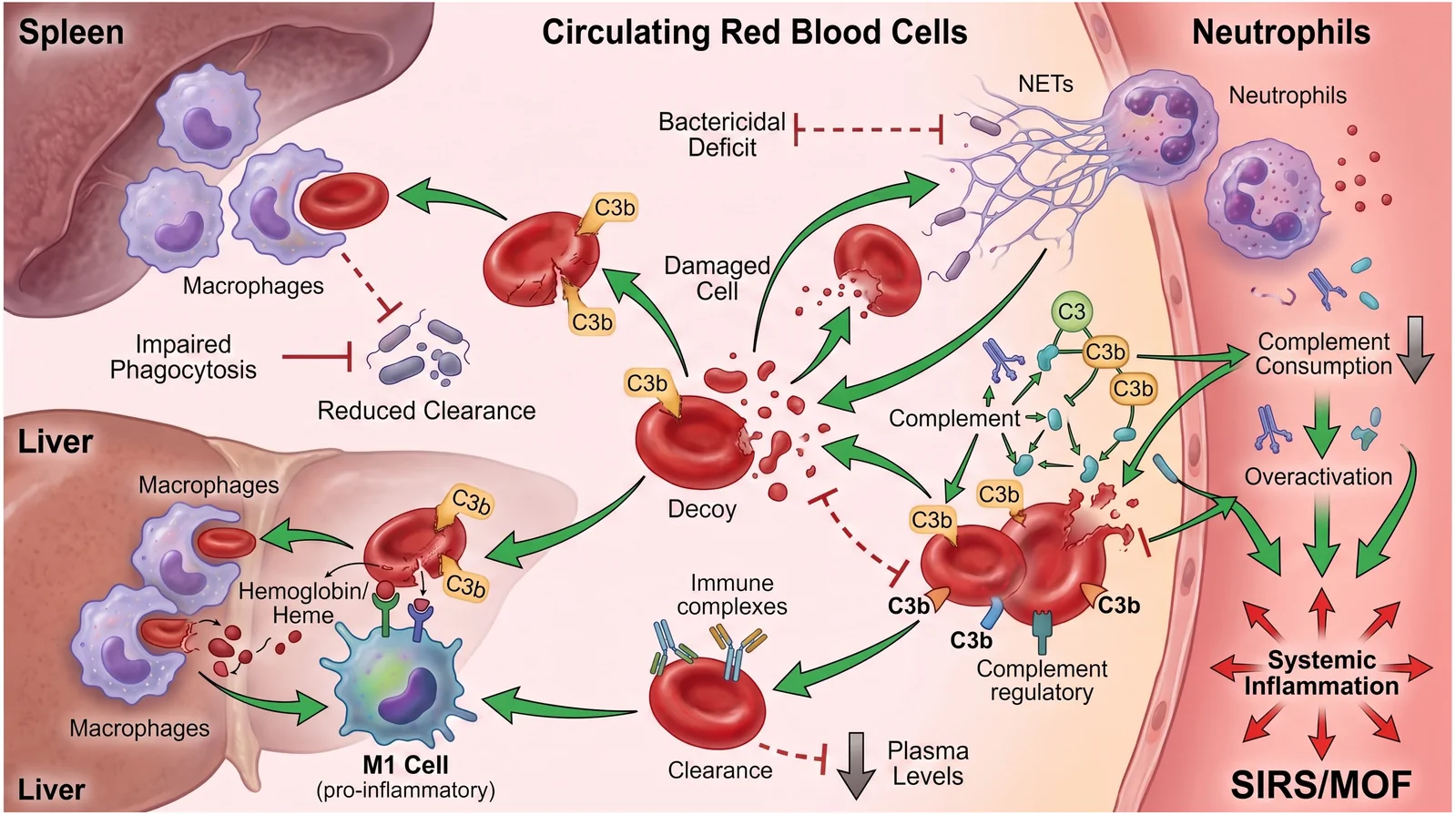
This schematic illustrates how erythrocytes interact with complement and phagocytes following trauma. Damaged erythrocytes expose surface ligands (e.g., PS) that promote phagocytic clearance by macrophages in the spleen and liver. Complement opsonins (C3b) deposited on erythrocytes enhance their recognition and clearance, while complement regulatory proteins limit overactivation. However, excessive complement activation may lead to complement consumption, impaired phagocytic function, and reduced clearance of pathogens and debris. Erythrocytes also bind circulating immune complexes via CR1 and transport them to phagocytes. Dysregulation of these pathways may amplify systemic inflammation, contributing to SIRS and multi-organ failure. Solid arrows: established pathways. Dashed arrows: pathways supported by trauma evidence. PS, phosphatidylserine; CR1, complement receptor 1; SIRS, systemic inflammatory response syndrome; MOF, multiple organ failure.

Direct evidence that the phagocytic system becomes functionally saturated after trauma is lacking, and whether such saturation contributes to impaired pathogen clearance in injured patients remains speculative (**Figure 1**).

### 3.2 Crosstalk with the complement system

Following trauma, complement activation creates a bidirectional interplay with erythrocytes, which serve both as targets and as regulators of complement activity. Erythrocytes express complement regulatory proteins on their surface, including decay-accelerating factor (CD55) and membrane inhibitor of reactive lysis (CD59), which protect host cells from inadvertent complement damage (24). Deposition of complement opsonins, such as C3b, on the erythrocyte surface promotes recognition and phagocytic clearance by macrophages in the reticuloendothelial system (38). Erythrocytes also bind circulating immune complexes via complement receptor 1 (CR1) and facilitate their transport to phagocytes, reducing immune complex burden and limiting complement activation.

Direct evidence from trauma patients demonstrates significant complement deposition on erythrocytes. Muroya et al. found that erythrocytes from trauma patients display significantly higher amounts of C4d on their surface compared with healthy controls, and trauma sera promote C4d deposition on donor erythrocytes (39). Complement-decorated erythrocytes exhibit limited deformability, accelerated calcium influx, and enhanced nitric oxide production. Trauma patients exhibit significant alterations in complement regulation. A recent multiomics study demonstrated that trauma patients with type O blood have decreased lectin pathway complement levels compared with other blood types (40), suggesting that genetic variation may influence individual susceptibility to complement-mediated pathology after injury. Excessive complement activation targeting erythrocytes, or impaired regulatory function, can lead to complement overconsumption and collateral tissue injury (24). In hemolytic conditions, erythrocyte ghosts are cleared by macrophage phagocytosis, a process facilitated by ubiquitin-proteasome-mediated degradation of CD47 on the ghost membranes, with the E3 ubiquitin ligase MARCH1 identified as a regulator (38). Efficient clearance of these hemolytic remnants is important for maintaining post-hemolytic immune homeostasis. The extent to which complement dysregulation on erythrocytes contributes to systemic inflammatory response syndrome and multi-organ dysfunction after trauma remains incompletely characterized. Most mechanistic insights derive from hemolytic disorders and transplantation models (26), with limited direct evidence from trauma populations.

## 4 Erythrocytes and the coagulation-inflammation crosstalk

### 4.1 Impact on platelet activation and thrombosis

Damaged erythrocytes release platelet activators, including adenosine diphosphate (ADP) and phosphatidylserine (PS)-exposing membranes, which may promote platelet aggregation and thrombosis. The supernatant from stored packed erythrocytes activates platelets, as evidenced by increased platelet-derived microparticle release, P-selectin expression, and integrin αIIbβ3 activation, an effect likely mediated by free hemoglobin and its breakdown products (25). In murine models, intravascular hemolysis triggers ADP-dependent platelet activation leading to precapillary pulmonary arteriolar thrombosis (41).

The procoagulant activity of erythrocytes is enhanced by PS externalization on their membrane. Phosphatidylserine is a pro-thrombotic phospholipid present within the erythrocyte cell membrane, and its externalization provides a catalytic surface for thrombin generation (42). In a pulmonary microcirculatory model, erythrocytes treated with tissue factor and calcium exhibit aggregation and accelerated thrombus formation, a process dependent on membrane PS.

Erythrocytes also interact with platelets through direct cell contact. A small population of erythrocytes localizes to platelet thrombi and enhances platelet activation via the FasL/FasR (CD95) pathway, which induces PS externalization on the erythrocyte membrane (43). During blood clot contraction, compression of erythrocytes activates mechanosensitive Piezo1 channels, leading to calcium influx, PS exposure, and subsequent amplification of platelet contractility (44).

Erythrocyte-derived microparticles (RMPs), released upon erythrocyte activation or injury, possess procoagulant activity. During extracorporeal membrane oxygenation—a setting involving mechanical erythrocyte stress—RMP levels are markedly elevated in patients with complications such as disseminated intravascular coagulation or severe hemolysis (45). Procoagulant microparticles are significantly elevated in trauma patients compared with healthy volunteers, and red cell-derived microparticles have been shown to induce a transient hypercoagulable state through accelerated activation of clotting factors in murine models (46). The relative contribution of erythrocyte-derived versus platelet-derived procoagulant signals in trauma-associated coagulopathy is an active area of investigation.

### 4.2 Involvement in endothelial dysfunction

Adhesion of erythrocytes to damaged endothelial cells may exacerbate endothelial barrier dysfunction and vascular hyperpermeability. Trauma-hemorrhagic shock induces rapid erythrocyte adhesion to endothelial cells in patients and animals via a CD36-dependent mechanism, which may explain the microvascular dysfunction occurring after trauma-hemorrhagic shock (47). The mechanical presence of adhered or aggregated erythrocytes can physically obstruct microvessels, contributing to ischemia. The activation of complement on erythrocytes compromises their deformability, further impairing microcirculatory flow (48).

Free hemoglobin, released during intravascular hemolysis, scavenges nitric oxide (NO), leading to vasoconstriction and impaired endothelium-dependent vasodilation (49). In trauma patients, intravascular hemolysis releases erythrocyte proteins such as hemoglobin and arginase-1; the latter disrupts arginine metabolism and limits physiologic NO production necessary to maintain organ perfusion (50). The loss of NO bioavailability removes a key anti-adhesive signal for platelets, promoting a prothrombotic phenotype. In hemolytic diseases, chronic hemolysis leads to sustained NO depletion, contributing to vasculopathic complications (51).

**Figure 2:**
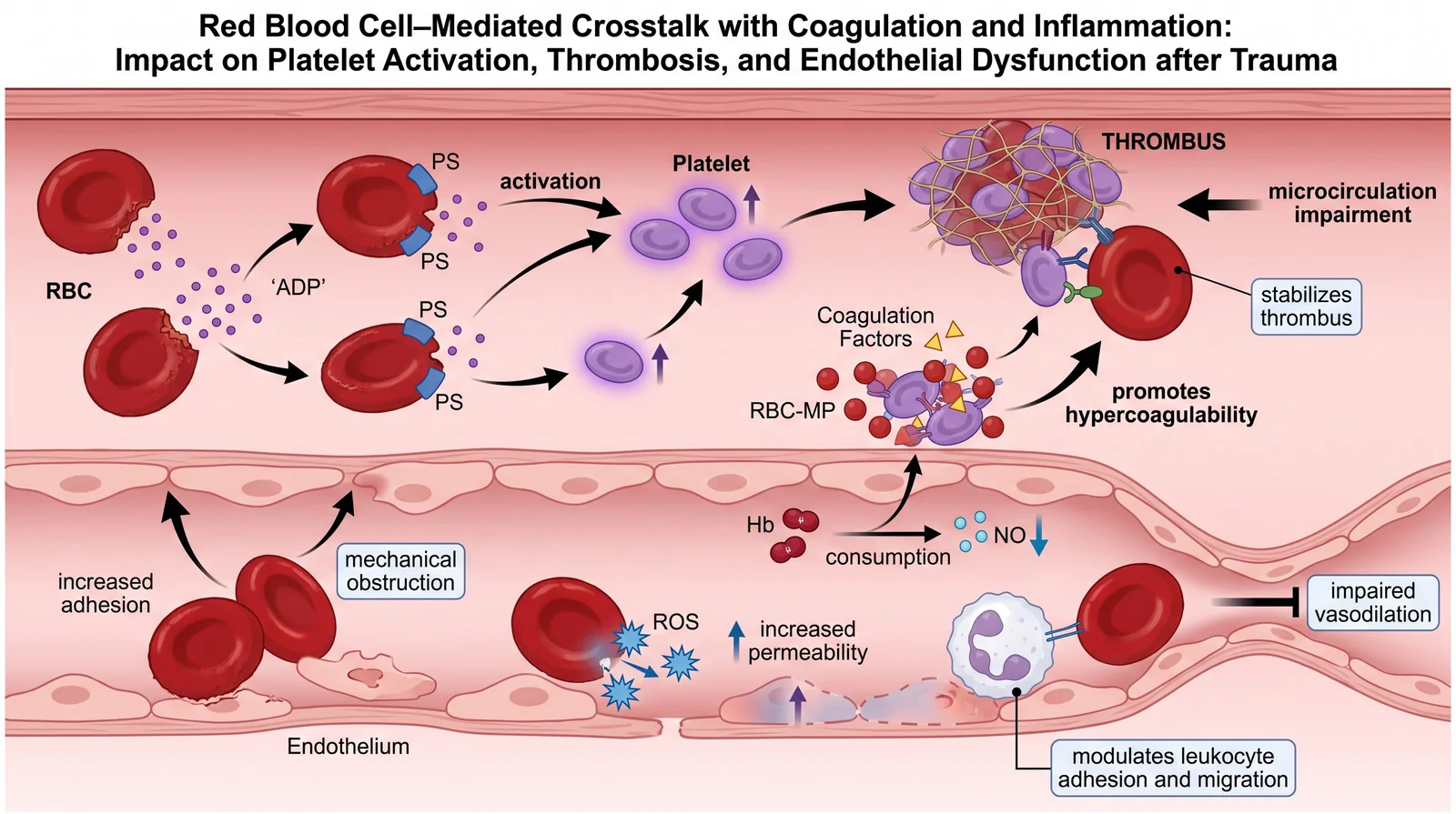
Red blood cells and the coagulation-inflammation crosstalk. This schematic illustrates how damaged erythrocytes promote a pro-thrombotic and pro-inflammatory state following traumatic injury. Phosphatidylserine (PS) exposure on damaged erythrocytes provides a catalytic surface for coagulation factor assembly and thrombin generation, activating platelets and promoting hypercoagulability. Erythrocyte-derived microparticles (RBC-MPs) carry procoagulant phospholipids that amplify thrombin generation. Hemolysis releases hemoglobin (Hb), which scavenges nitric oxide (NO), impairing vasodilation, while reactive oxygen species (ROS) increase endothelial permeability. Erythrocytes also adhere directly to endothelium via CD36, contributing to microvascular obstruction. These combined effects result in microcirculatory impairment, thrombus stabilization, and reduced tissue perfusion. PS, phosphatidylserine; RBC-MP, red blood cell-derived microparticle; Hb, hemoglobin; ROS, reactive oxygen species; NO, nitric oxide.

Interactions between erythrocytes and the endothelium may modulate the rolling, adhesion, and migration of inflammatory cells to the site of injury. Erythrocyte transfusion has been associated with enhanced endotheliopathy after trauma, and erythrocyte-derived extracellular vesicles may contribute to endothelial activation and injury (52). Heme-induced platelet activation leads to P-selectin expression on platelets, increasing platelet-neutrophil aggregate formation and subsequent NETosis, which further damages the endothelium and promotes thrombosis (53). The relevance of these endothelial interactions to acute trauma—as opposed to chronic hemolytic conditions—is supported by direct evidence from trauma patients demonstrating rapid erythrocyte-endothelial adhesion (47) and transfusion-associated endotheliopathy (52). However, the precise molecular mechanisms and their relative contribution to post-traumatic organ dysfunction require further elucidation (**Figure 2**).

## 5 Pathological consequences of erythrocyte dysfunction in trauma

### 5.1 Promotion of secondary organ injury

#### 5.1.1 Acute kidney injury

Intravascular hemolysis releases hemoglobin and heme, which contribute to acute kidney injury (AKI) through direct tubular toxicity, intrarenal vasoconstriction, and oxidative stress (54). Hemoglobin and heme promote AKI via proximal tubule epithelial cell injury and tubular cast formation. In trauma patients, elevated hemolysis markers are significantly associated with adverse outcomes including organ dysfunction, and patients with lower fibrinogen levels—often linked to hemorrhage and transfusion—have an increased risk of developing multiple organ failure and AKI (53). A single-center study of polytrauma patients reported an AKI incidence of 30.5%, with hemorrhagic shock, massive transfusion, and rhabdomyolysis identified as the primary risk factors (55). Transfusion practices themselves may contribute to renal injury; in children with life-threatening bleeding, an increased platelet-to-RBC transfusion ratio is independently associated with a higher risk of AKI. The toxic effects of free hemoglobin and heme involve the generation of reactive oxygen species that damage renal tubular cells and compromise microvascular flow.

#### 5.1.2 Acute lung injury

Erythrocyte dysfunction is implicated in the pathogenesis of acute lung injury (ALI) through mechanisms including neutrophil sequestration in pulmonary capillaries and increased vascular permeability. Cell-free hemoglobin increases leukocyte adhesion and initiates lung microvascular endothelial activation via Toll-like receptor 4 signaling. Heme-induced platelet activation promotes platelet-neutrophil aggregate formation and NETosis, leading to pulmonary damage. Transfusion of blood products, particularly RBCs, is a recognized risk factor for transfusion-related acute lung injury (TRALI). Stored RBCs develop “storage lesions,” accumulating bioactive mediators including mitochondrial DNA that act as DAMPs, activating innate immune pathways and leading to neutrophil activation, NET formation, and endothelial damage (56). In a murine model of TRALI, mitochondrial DNA via recipient TLR9 was identified as a potent priming “first-hit” that predisposes to antibody-mediated lung injury (57). A three-step pathogenesis model—priming, pulmonary reaction, and effector phase—has been proposed to explain the progression from recipient predisposition to end-organ injury in TRALI (58). Patients receiving liberal RBC transfusion strategies have an increased risk of developing acute lung injury compared with those on restrictive strategies (59).

#### 5.1.3 Distal organ dysfunction and microcirculatory disturbance

**Figure 3:**
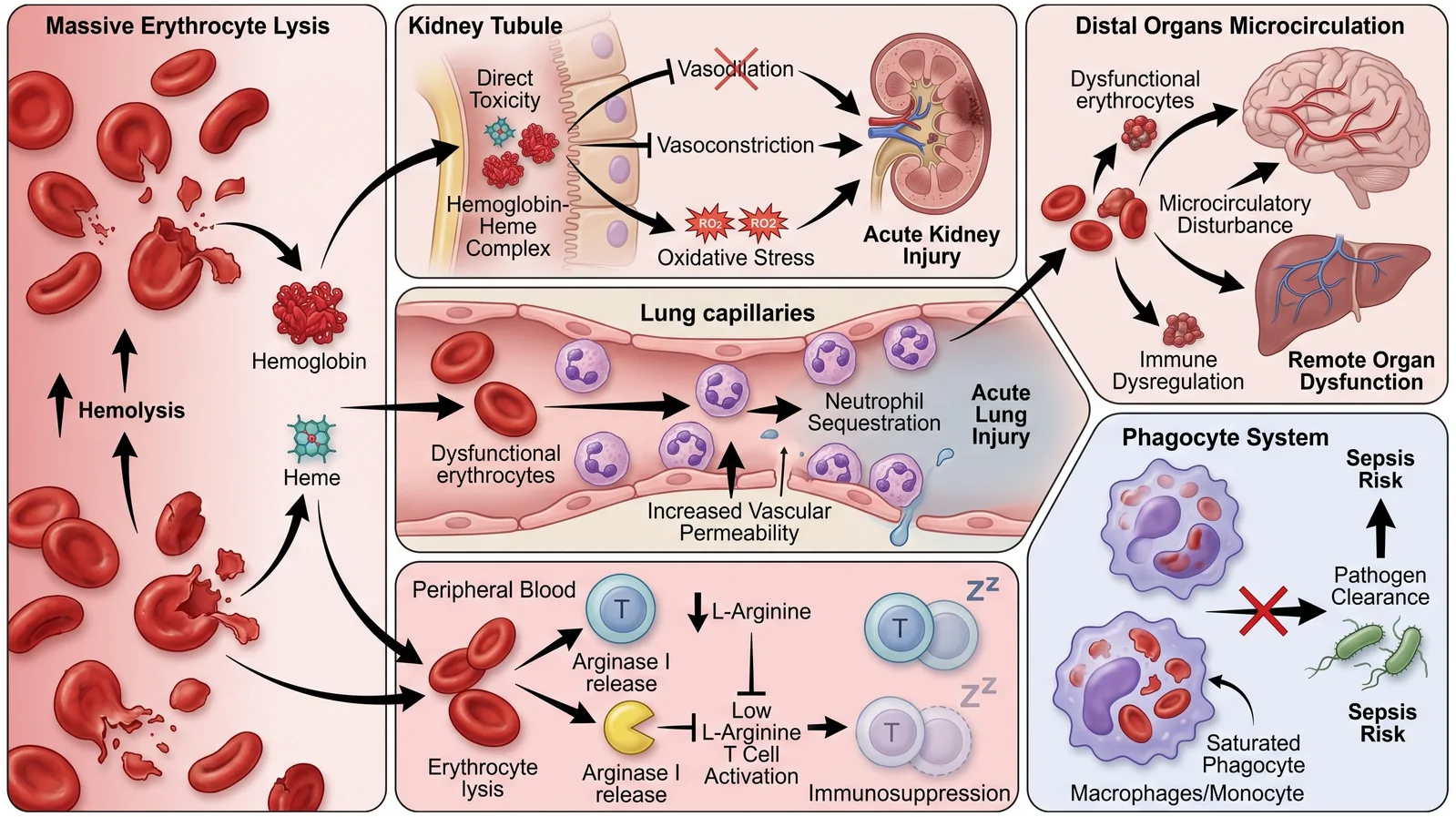
This schematic illustrates how erythrocyte dysfunction following trauma leads to secondary organ injury, immunosuppression, and increased infection susceptibility. Massive erythrocyte lysis releases hemoglobin and heme, which cause direct tubular toxicity, oxidative stress, and vasoconstriction/dysfunctional vasodilation, contributing to acute kidney injury. Hemoglobin and heme also promote neutrophil sequestration in pulmonary capillaries, increasing vascular permeability and leading to acute lung injury. Hemolysis releases arginase I, which depletes L-arginine, impairing T-cell function and promoting immunosuppression. Erythrocyte fragments saturate the phagocytic system, reducing pathogen clearance and increasing sepsis risk. Microcirculatory disturbances and immune dysregulation contribute to distal organ dysfunction.

Erythrocyte-mediated immune dysregulation and microcirculatory disturbances constitute a pathological basis for distal organ dysfunction, including post-traumatic brain and liver injury (60). Following severe trauma, systemic hemolysis and RBC transfusion can trigger immune dysregulation and impair microvascular flow. In trauma patients, mononuclear cell arginase activity rises early after injury, coinciding with decreased plasma arginine and increased IL-10 levels. Arginase-1 released from erythrocytes contributes to L-arginine depletion and subsequent reduction in nitric oxide production necessary for organ perfusion (61). This metabolic dysregulation can suppress T-cell function and impair organ recovery. Microvascular thrombosis causes local ischemia and releases RBC debris, fueling further inflammation and endothelial injury—a cycle that underpins multiple organ dysfunction syndrome. High transfusion volumes (ultramassive transfusion) are associated with increased rates of multiple organ failure in trauma patients (62) (**Figure 3**).

### 5.2 Induction of immunosuppression and susceptibility to infection

#### 5.2.1 T-cell dysfunction and immunosuppressive mechanisms

Excessive early post-traumatic erythrocyte clearance and the accompanying inflammatory response may lead to immune cell exhaustion and dysfunction, precipitating a state of immune paralysis. Following major trauma, there is massive activation and consumption of innate immune cells in response to tissue damage and hemolysis. Mitochondrial damage-associated molecular patterns (mitoDAMPs) released from injured cells induce NK cells and T cells to adopt regulatory phenotypes; T cell proliferation is interrupted in the presence of mitoDAMPs, and this effect is mediated by arginase-dependent arginine depletion (63). In anemic states, CD71+ erythroid cells expand and express arginase 1 and 2, which suppress T-cell IFN-γ production and proliferation through L-arginine depletion (64). While these responses may limit excessive inflammation, they also create vulnerability to secondary infections. The burden of erythrocyte debris may further overwhelm phagocytic capacity.

#### 5.2.2 Macrophage dysfunction and phagocytic saturation

Saturation of the reticuloendothelial system with erythrocyte fragments may impair macrophage clearance of pathogens. In sepsis models, acute stress erythrophagocytosis induces an immunosuppressive macrophage phenotype through heme-mediated STAT1 dysregulation (65). Following massive hemolysis or transfusion, physical occupation of phagocytic capacity by erythrocyte debris, together with heme- and iron-driven functional reprogramming, may induce an anti-inflammatory macrophage phenotype less effective at bacterial killing. Consequently, when new bacterial challenges arise, the phagocytic system responds poorly, increasing bloodstream infection and sepsis risk (**Figure 3**).

## 6 Therapeutic implications and clinical translation of erythrocyte-targeted approaches

### 6.1 Strategies to mitigate erythrocyte injury

Cell-free hemoglobin and heme released from hemolyzed erythrocytes are potent mediators of endothelial dysfunction, organ injury, and immunomodulation in trauma. Physiologic scavenging systems—haptoglobin for hemoglobin and hemopexin for heme—are overwhelmed when hemolysis is extensive, as occurs in major trauma and massive transfusion. Therapeutic supplementation of these scavengers, along with antioxidant and iron-chelating approaches, has been explored in preclinical models to restore clearance capacity and limit hemolysis-driven tissue injury (66).

Complement dysregulation also contributes to erythrocyte destruction and DAMP release. Anti-C5 monoclonal antibodies have been used successfully in complement-mediated hemolytic conditions such as thrombotic microangiopathy (67). In a rat model of penetrating traumatic brain injury, intravenous anti-CD47 antibody augmented hematoma clearance, mitigated acute neuropathology, and improved cognitive function (68). This proof-of-concept study suggests that enhancing phagocytic clearance of erythrocytes from hematomas may reduce secondary injury, though systemic application raises concerns about off-target effects on viable erythrocytes.

### 6.2 Erythrocyte-based therapeutic platforms

The CD47-SIRPα axis has been explored not only for hematoma clearance but also for modulating immune responses. However, systemic blockade carries risks of anemia and altered immune homeostasis, as CD47 is expressed on all viable erythrocytes. Supra-prophylactic doses of enoxaparin have also been investigated for reducing fibrin deposition without exacerbating hemorrhage in TBI models (69), suggesting that anticoagulant strategies may be feasible in selected trauma populations.

Erythrocytes. and RBC membrane-derived nanoparticles have been developed as bioinspired drug delivery systems to address issues of premature clearance, toxicity, and immunogenicity. RBC-based delivery systems possess characteristics including biocompatibility, biodegradability, and long circulation time (70, 71). Whole RBCs, RBC membrane-camouflaged nanoparticles, RBC-derived extracellular vesicles, and RBC hitchhiking strategies have all been explored. RBC membrane-coated nanoparticles have been designed for targeted delivery—for example, prednisolone acetate-loaded PLGA nanoparticles for kidney targeting (72) and RGD peptide-modified nanoparticles for thrombolytic therapy (73). While most applications have been developed in oncology and autoimmune models, the principles are transferable to trauma, such as targeting anti-inflammatory agents to injured tissues or delivering pro-resolving mediators to sites of organ injury.

### 6.3 Clinical translation: implications for trauma management

The landmark HEMOTION trial, published in the New England Journal of Medicine (2024), randomly assigned adults with moderate or severe traumatic brain injury and anemia to receive RBC transfusion according to a liberal strategy (transfusion initiated at hemoglobin ≤10 g/dL) or a restrictive strategy (transfusion initiated at ≤7 g/dL), finding that a liberal strategy did not reduce the risk of an unfavorable neurologic outcome at 6 months (74). A systematic review and meta-analysis published in Annals of Intensive Care (2024) further confirmed that restrictive transfusion strategies did not reduce mortality or unfavorable neurological outcomes compared with liberal strategies in TBI patients (75). Transfusion kinetics—rate and timing of administration—may impact the inflammatory response beyond total transfusion volume, suggesting that future protocols might be refined to minimize immunomodulatory complications.

Several RBC-related biomarkers show promise for identifying harmful hemolysis and dysfunction after trauma. Elevated plasma cell-free hemoglobin (CFH) has been identified as a novel early biomarker following traumatic injury. In a prospective study of 119 severely injured trauma patients published in the Journal of Trauma and Acute Care Surgery (2025), CFH was found to be significantly elevated upon emergency department arrival (median 10.9 μM; IQR, 6.8–17.6) and remained elevated compared with normative levels during the monitoring period. Patients with the highest CFH levels (median 25 μM) had longer hospital stays and more frequent complications, suggesting that early correction of hemolysis could provide clinical benefit (76). A scoping review in Critical Care Explorations (2024) confirmed that CFH is a potent mediator of endothelial dysfunction, organ injury, coagulopathy, and immunomodulation in hemolysis (66). Elevated extracellular particle (EP) concentrations, particularly extracellular vesicles <200 nm, predict in-hospital mortality after severe trauma. In a study of 133 severely injured patients (ISS ≥16) published in Frontiers in Immunology (2024), an optimal cut-off of 17,380/μl plasma demonstrated 77% sensitivity and 61% specificity in predicting in-hospital mortality; non-survivors received significantly higher numbers of packed RBC units compared with survivors (77). Arginase-1, an enzyme released from hemolyzed erythrocytes, has been identified as a potential mediator of immunosuppression through arginine depletion (61); trauma is associated with decreased nitric oxide metabolite production and a state of immunosuppression characterized by increased IL-4 production (51). Complement deposition (C4d) on erythrocytes has been demonstrated in trauma patients and correlates with reduced deformability (78), suggesting utility as a marker of complement-mediated injury.

Challenges to clinical translation include the lack of point-of-care assays for most markers, difficulty distinguishing native trauma-induced injury from transfusion-related effects, and heterogeneity of trauma populations that complicates biomarker validation. Prospective studies are needed before routine implementation can be recommended.

## 7 Discussion

The mechanisms reviewed in this manuscript—surface remodeling, DAMP release, complement activation, phagocyte modulation, and endothelial interaction—share a common origin: the damaged erythrocyte. Erythrocytes constitute approximately 25% of all human cells, and their membrane surface area exceeds that of all endothelial cells combined (79). This quantitative reality explains why erythrocyte-derived signals appear in nearly every pathway linking trauma to organ injury and immune suppression. A unifying observation is that many of these pathways converge on TLR4 and TLR9 signaling (80). Heme activates TLR4; mtDNA activates TLR9. Both trigger NF-κB-dependent inflammation, promote platelet activation, and impair endothelial barrier function. This convergence suggests that the clinical syndrome of post-traumatic organ dysfunction—manifesting as simultaneous AKI, ALI, and coagulopathy—may represent a single underlying process driven by erythrocyte-derived DAMPs, rather than multiple independent organ-specific injuries (81). The release of DAMPs from erythrocytes through programmed cell death pathways further amplifies this inflammatory cascade.

The dual nature of erythrocyte responses appears to depend on three factors: injury severity, duration of hemolysis, and clearance efficiency. Early after trauma, low-level DAMP signaling may mobilize innate immunity (82). When hemolysis is massive or sustained, the same signals overwhelm clearance systems and drive immunoparalysis. This temporal dimension is rarely captured in current studies, yet it may be the key to therapeutic timing. A paradox emerges from the clinical data: erythrocyte dysfunction correlates with poor outcomes, yet erythrocyte transfusion can exacerbate inflammation and immunosuppression (53, 83). This paradox is likely explained not by the erythrocytes themselves but by storage lesions, transfusion kinetics, and the inflammatory state of the recipient. The implication is that the goal is not merely to increase erythrocyte count, but to optimize erythrocyte quality, timing, and immune context.

## 8 Conclusion

This review has examined the emerging roles of erythrocytes in traumatic immunopathology, emphasizing that they are not merely victims of trauma but active participants in determining clinical outcomes. The evidence indicates a dual role for erythrocytes in trauma: beneficial homeostatic functions can become maladaptive when dysregulated, driving thrombo-inflammation, organ injury, and immunosuppression. This duality informs both the challenges and opportunities for therapeutic intervention—whether through neutralizing toxic byproducts, modulating clearance pathways, or engineering erythrocyte-based delivery systems.

Translation of these insights from bench to bedside requires continued investigation in human trauma cohorts, refinement of therapeutic strategies targeting erythrocyte pathways, and integration of erythrocyte functional assessments into clinical management. By elucidating the erythrocyte’s central role in trauma pathophysiology, this work charts a course toward novel diagnostic and therapeutic avenues with the potential to improve outcomes for trauma patients globally. A summary of the key pathways, their supporting evidence, and the major knowledge gaps is provided in the following table (**Table 1**).

**Table 1:** Erythrocyte mechanisms in trauma.

Pathway	Key event	Direct trauma evidence	Indirect evidence	Clinical relevance	Knowledge gap
Surface Remodeling	PS externalization; CD47 downregulation	ssPS+ RBCs not systematically quantified in trauma cohorts	ECLS: mechanical stress induces PS exposure; polycythemia models: CD47-SIRPα blockade reverses phenotype	Modulates RBC clearance rate, macrophage function, and anemia severity	PS/CD47 dynamics on circulating RBCs after trauma remain uncharacterized
DAMP Release	Heme, hemoglobin, mtDNA release into plasma	Trauma patients: elevated plasma heme correlates with poor outcomes; mtDNA detected	Subdural hematoma: RBC lysate drives TLR4-dependent neuroinflammation	Triggers SIRS, endothelial activation, and remote organ injury	Relative contribution of RBC-derived vs. tissue-derived DAMPs unknown
Complement Regulation	C3b/C4d deposition; CR1-mediated clearance	Trauma patients: C4d on RBCs; complement-decorated RBCs show reduced deformability; type O blood shows decreased lectin pathway levels	TMA: complement dysregulation drives RBC destruction and organ injury	Regulates RBC clearance, complement consumption, and tissue injury	Functional consequences of complement deposition on RBCs after trauma undefined
Phagocyte Modulation	Erythrophagocytosis; HMGB1-mediated suppression	Phagocyte dysfunction inferred from anemia and infection patterns	Sterile inflammation: Hb/heme induce M1, erythrophagocytosis induces M2; trauma-hemorrhagic shock model: stored RBC supernatant inhibits macrophage phagocytosis via HMGB1	Affects macrophage phenotype and pathogen clearance capacity	Whether phagocytic system becomes functionally saturated after trauma unknown
Platelet & Thrombosis	PS/ADP exposure; RBC-MP release	RBC procoagulant activity inferred from trauma coagulopathy patterns	Murine models: hemolysis triggers ADP-dependent platelet activation; trauma models: PS-dependent thrombus formation	Promotes microvascular thrombosis and contributes to MODS	Causal role of RBC-derived MPs in trauma coagulopathy requires investigation
Endothelial Dysfunction	RBC adhesion; NO scavenging; ROS generation	Trauma patients: CD36-dependent RBC-endothelial adhesion demonstrated; hemolysis disrupts arginine-NO metabolism	SCD: chronic hemolysis leads to NO depletion and vasculopathy; subdural hematoma: RBCs drive neuroinflammation	Causes vasoconstriction, impaired perfusion, and endotheliopathy	Systemic endothelial interactions in acute trauma poorly characterized
Organ Injury	Hb/Heme tubular toxicity; pulmonary permeability	Trauma patients: hemolysis markers associated with AKI/MODS; polytrauma: AKI incidence 30.5%; liberal transfusion increases ALI risk	Rhabdomyolysis: hemolysis biomarkers associated with AKI	Links hemolysis to AKI, ALI, and multiple organ dysfunction	Relative contribution of hemolysis vs. other injury factors to organ failure unclear
Immunosuppression	Arginase I release; L-arginine depletion	Trauma patients: mononuclear cell arginase activity elevated; correlates with low arginine and high IL-10; low arginine associates with poor outcomes	Trauma models: MDSCs express arginase 1, infiltrate spleen within 6 hours; mitoDAMP triggers arginase-dependent T-cell suppression	Drives T-cell dysfunction, immune paralysis, and infection susceptibility	Whether arginase-mediated immunosuppression is therapeutically targetable unknown
Phagocytic Saturation	RBC fragments overwhelm phagocytic capacity	Indirect (phagocyte dysfunction observed in severely injured patients)	Sepsis models: stress erythrophagocytosis induces immunosuppressive macrophage phenotype via heme-STAT1 dysregulation	Increases bloodstream infection and sepsis risk	Clinical significance of phagocytic saturation in trauma patients unknown
